# Unveiling the Physiological Correlates of Cognitive Function in Healthy Adults: An In-Depth Investigation Using Optical Coherence Tomography (OCT)

**DOI:** 10.3390/jcm15020496

**Published:** 2026-01-08

**Authors:** Sarah Al-Mazidi, Hanan Khalid Mofty, Kholoud Ahmad Bokhary, Najla Mohammed ALdughayshim, Laila Al-Ayadhi, Adel G. Alakeely

**Affiliations:** 1 Anatomy and Physiology Department, College of Medicine, Imam Mohammad Ibn Saud Islamic University, Riyadh 13318, Saudi Arabia; 2 Optometry Department, College of Applied Medical Sciences, King Saud University, Riyadh 12372, Saudi Arabia; 3 Addiction Treatment and Recovery Center—Qawim, Riyadh 12836, Saudi Arabia; 4 Autism Research and Treatment Center, King Saud University, Riyadh 11461, Saudi Arabia; 5 Department of Physiology, Faculty of Medicine, King Saud University, Riyadh 11461, Saudi Arabia; 6 Magrabi Hospital and Center, Riyadh 13241, Saudi Arabia

**Keywords:** cognition physiology, cognitive impairment, retina, OCT, cognitive screening

## Abstract

**Background/Objectives**: The search for biomarkers of cognition has garnered significant interest over the past decade, owing to their objective nature, in contrast to the currently available cognition screening tools, which are based on subjective measures. Retina imaging is used in this field because its tissue is considered as an extension of the brain’s vascular and neural structures, reflecting overall brain health. In cognitive disorders, early detection and intervention are essential for achieving the best possible outcomes. To evaluate the physiological correlates of cognitive function in healthy young adults by assessing retinal structures as a non-invasive biomarker of cognitive health. **Methods**: Eighty healthy young adults participated in this study. Optical Coherence Tomography (OCT) was used to measure retinal morphology, including macular thickness, volume, and retinal nerve fiber layer thickness; then, OCT results were correlated with cognitive assessments using the Montreal Cognitive Assessment (MoCA). **Results**: Participants with mild cognitive impairment exhibited thinner macular thickness and lower macular volume (*p* < 0.05, *p* < 0.001) than participants with normal cognitive function. We also found that macular thickness is positively associated with cognitive function in healthy adults (*p* < 0.001). The RNFL was found to be normal in all groups, despite changes in macular thickness, indicating that cognitive function in normal individuals depends on macular changes rather than the optic nerve. **Conclusions**: Macular OCT, which is a cost-effective and widely available tool, can be used to screen for mild cognitive impairment. A clinical trial is recommended to validate these findings and to generate guidelines for assessing cognitive physiology through the retina.

## 1. Introduction

There is great interest in identifying the biomarkers of cognitive function that predict cognitive impairment and the risk of future dementia. Different biomarkers were deployed, but they are generally considered inconvenient, expensive, and time-consuming. These include neuroimaging, blood or cerebrospinal fluid samples [1,2]. Recently, there has been a growing interest in diagnosing neurodegenerative disorders and cognitive function through the eye [3].

The retina can be considered an extension of the brain, composed of neuronal and glial elements similar to those of cerebral architecture. Retinal imaging provides a unique opportunity to study brain-like structures in vivo, with Optical Coherence Tomography (OCT) of the retina and optic nerve providing a microstructural, cross-sectional, and quantitative analysis of the eye’s tissues, allowing visualization of distinct internal layers with near-histologic resolution [4]. Compared to other imaging techniques such as Positron Emission Tomography (PET) and Magnetic Resonance Imaging (MRI), OCT is faster, cheaper, widely available, and involves no radiation or invasive procedures. It can reveal fine structural changes in the retina that may reflect early neurodegenerative processes before clinical symptoms arise [5]. Notably, the retina contains discrete layers with different functional and anatomical significance. The photoreceptor layer is essential for converting light into neural signals. The neuronal bodies and axons of the retinal ganglion cell layer (GCL), along with the Retinal Nerve Fiber Layer (RNFL), are connected to the visual pathways in the central nervous system. The inner retinal layers, consisting of the ganglion cell layer (GCL) and the inner plexiform layer (IPL), were found to be affected in neurodegenerative disease, specifically Alzheimer’s disease [6]. Changes in the retina were also found in Multiple sclerosis, in which the authors found that zonal thinning arises from trans-synaptic and synaptic degeneration, as well as from immune-mediated axonal injury with secondary retrograde neuronal loss [7]. These OCT parameters have shown significant associations with disease status and progression of previously diagnosed patients with Alzheimer’s disease, Lewy body dementia, multiple sclerosis, and other neurological disorders [7,8,9]. Also, retinal OCT changes were observed in mild cognitive impairment before the development of Alzheimer’s disease [10,11].

Previous studies examined cognitive function through the retina in patients with neurological disorders, but studies in young, healthy adults are scarce. Testing the retina of a normal young adult would provide an opportunity to understand the relationship between the retina and cognitive function without the effects of aging and neurological dysfunction, which might affect retinal health. Abnormalities in retinal thickness in young and healthy adults typically do not affect vision. Hence, they do not generally present for eye exams, which may be one reason retinal thickness in the early stages of cognitive or neurodegenerative illnesses is not well documented.

This study evaluates the physiological correlates of cognitive function in a cohort of healthy young adults by assessing retinal structures through retinal and optic nerve OCT. We aim to determine whether variations in macular thickness, volume, and RNFL thickness relate to performance on cognitive assessments, and to assess the potential of retinal morphology as a non-invasive biomarker for cognitive health.

## 2. Materials and Methods

This study was conducted in the Optometry department at the Applied Medical Clinics at King Saud University. Institutional Review Board and Guidelines of Health Sciences Colleges Research on Human Subjects, King Saud University, College of Medicine, approved this study with relevant guidelines in accordance with the Declaration of Helsinki (Ref. No. 24/0008/IRB-A). Informed consent was obtained from all participants before they approved the processing and publication of their data.

### 2.1. Participants

Eighty participants were initially screened to participate in this study (160 eyes). Demographic data, including age, gender, and education, were obtained from each participant. Medical data were also collected from all participants, including current and previous illnesses and regular medications. Healthy adults of both genders aged 18 to 35 met the inclusion criteria. Participants with known or family history of neurological or mental conditions or those using regular medications of any kind were excluded. Also excluded were patients with Ocular diseases such as glaucoma or acquired or inherited retinopathies, and high refractive error (hyperopia ≥ +6D sphere, myopia ≥ −6D sphere, ≥+3D cylinder, amblyopia, axial refractive error).

All patients were interviewed for demographic and medical data, then signed a consent form to participate and underwent testing for cognitive and retinal function. Participants were excluded according to predefined criteria, determined through consensus review by a neurophysiologist, ophthalmologists, and researchers, based on clinical assessments and OCT imaging.

### 2.2. Cognitive Function

The Montreal Cognitive Assessment (MoCA) was used to assess cognitive function in our study population. The test was administered by a certified interviewer who guided participants through it. It is a validated, rapid screening tool that assesses the main cognitive domains, including visuospatial/executive function, naming, memory, attention, language, abstraction, calculation, and orientation [12]. The visuospatial domain tests the ability to perceive and process visual information, including trail-making and copying a graph (score 5). Naming involves displaying images and asking participants to name these images (score 3). The memory domain tests short-term memory by naming five objects, and the delayed recall test assesses recall of these five objects by the end of the test (score 5). Attention includes naming a string of numbers as they are said by the examiner (score 6). Language domain includes repeating a sentence (score 3). The abstraction domain tests the ability to find a relationship between two words (score 2). Finally, the orientation domain test assesses the ability to state the current date, time, and clinic location (score 6). Certified personnel performed this test face-to-face for all participants. It consists of 30 points; a score of 26 or higher is considered normal. A score between 25 and 18 is considered mild cognitive impairment, a score of 17 to 10 is considered moderate cognitive impairment, and a score below 10 is considered severe cognitive impairment.

### 2.3. Optical Coherence Tomography (OCT)

All participants underwent high-resolution spectral-domain optical coherence tomography (SD-OCT) imaging using the Cirrus 6000 HD-OCT system (Carl Zeiss Meditec, Dublin, CA, USA) to evaluate structural features of the macula and optic nerve head (ONH). Imaging was performed using standardized acquisition protocols. For macular assessment, the 512 × 128 macular cube scan (covering 6 × 6 mm) centered on the fovea was used. Key parameters included central subfield thickness (CST), average macular thickness, and total macular volume. The macular thickness (measured from the inner limiting membrane to the retinal pigment epithelium) was displayed on a screen using the ETDRS grid format. The ETDRS grid consists of three rings: a central circular subfield with a diameter of 1 mm around the fovea, an inner parafoveal ring with a diameter of 3 mm, and an outer perifoveal ring with a diameter of 6 mm. The inner and outer rings are each divided into four quadrants: superior, inferior, temporal, and nasal. Optic nerve head (ONH) imaging was performed using the 200 × 200 optic disc cube scan protocol to obtain peripapillary retinal nerve fiber layer (RNFL) thickness measurements. Outcomes included average RNFL thickness and quadrant-specific thickness values (superior, inferior, nasal, temporal). One masked trained grader, masked to participant characteristics, reviewed OCT datasets, excluding those with poor quality (scan signal strength, excessive movement artefacts, or inconsistent signal intensity across the scan) or missing variables.

OCT parameters were compared between individuals with normal and abnormal cognitive testing results. The primary OCT outcomes included CST and macular volume, and average RNFL thickness and quadrant-specific RNFL thickness for the ONH.

### 2.4. Statistical Analysis

The data were analyzed using IBM SPSS Statistics (Version 27). Data were presented as mean ± standard deviation (SD). Data that are not normally distributed (as determined by the Shapiro–Wilk normality test) are presented as medians. The Student’s *t*-test was used to compare retinal layers in the normal cognitive function group and the moderate cognitive impairment group. The Mann–Whitney U test was used for data that were not normally distributed. A linear regression analysis was conducted to determine the relationship between cognitive function scores and retinal layer thickness. Two-tailed tests for statistical significance were performed, with *p* < 0.05 considered significant. Statistical significance was denoted by * *p* < 0.05, ** *p* < 0.01, and *** *p* < 0.001.

## 3. Results

### 3.1. Demographics

Of the 91 participants, 11 patients were excluded due to incomplete data, high refractive errors, or poor-quality OCT. A total of 80 participants (160 eyes) were included in our study (43 females and 37 males). The mean age was 23.08 ± 4.1 years. All participants had a similar educational background.

### 3.2. Cognitive Function Analysis

The mean cognitive function, as measured by the MoCA, was 25.47 ± 5.3. There were 38.8% participants with mild cognitive impairment, with a mean of 23.47 ± 1.3, and 61.2% with normal cognitive function, with an average of 27.9 ± 1.3. Table 1 shows the average scores for different aspects of the MoCA test, including visuospatial/executive function, naming, memory, attention, language, abstraction, calculation, and orientation. A perfect score was granted for all participants in the naming section. The lowest scores were found mainly in the visuospatial (3.93 ± 1.2/5) and delayed recall (3.59 ± 1.5/5) sections. Table 2 shows the differences in MoCA domains between individuals with mild cognitive impairment and those with normal cognitive scores. A significant difference was observed in the visuospatial and delayed recall domains between the two groups (*p* < 0.05 and 0.01, respectively).

### 3.3. Retinal Structure

The Optical Coherence Tomography (OCT) findings are demonstrated in Table 3. The average retinal thickness was 275.5 ± 19.7 µm, the central thickness was 241.24 ± 34.6 µm, and the volume was 9.14 ± 1.4 mm^3^. Also, the average retinal nerve fiber layer (RNFL) thickness in OCT was 96.53 ± 9.7 µm in the normal cognitive group and 96.27 ± 8.5 µm in the mild cognitive impairment group. The OCT analysis between both genders showed that the average retinal thickness was 269.6 ± 13.5 µm in female participants and 282.2 ± 11.9 µm in male participants, and that the average RNFL thickness in females was 95.74 ± 10.6 µm and in males was 96.47 ± 8.6 µm.

The results of the OCT were compared with MoCA scores within the population studied. Lower scores on cognitive testing were associated with significantly reduced macular thickness, despite RNFL thickness being preserved in the mild cognitive impairment group compared with the normal cognitive function group (*p* < 0.05). Additionally, a significant decrease in total macular volume was observed in the mild cognitive impairment group (*p* < 0.001). Notably, thinning was most pronounced in the parafoveal region, particularly in the nasal and temporal quadrants, highlighting the central macular zones as potentially sensitive indicators of subtle cognitive variation.

**Figure 1:**
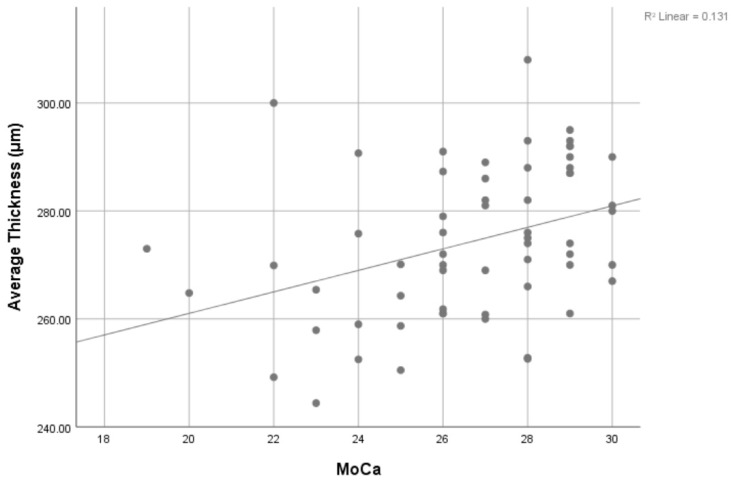
Regression analysis showing a positive relationship between retinal average thickness (µm) and MoCA score. There is a (0.076) increase in average thickness of the retina for every (1) increase in the MoCA score (*p* < 0.001).

Linear regression analysis (Figure 1) showed that average retinal thickness increased with increasing MoCA scores (R = 0.078). The OCT results for the retinal nerve were similar between the mild cognitive impairment group (95.27 ± 8.5) and the normal cognitive function group (95.53 ± 9.7).

## 4. Discussion

This study demonstrated the relationship between cognitive function and retinal structural changes, including macular thickness and volume, and the retinal nerve fiber layer (RNFL), in the normal population. This supports the hypothesis that retinal morphology, particularly in the macular region, may be a physiological biomarker of cognitive function in healthy individuals. A significant relationship was found between cognitive function and macular layer, average thickness, and volume. Contrary to findings in Alzheimer’s disease, where RNFL thinning is commonly reported, RNFL values in this young, healthy cohort did not correlate with cognitive function, suggesting that RNFL thinning may be a late-stage marker in the cognitive decline trajectory [10,11]. Instead, early changes may manifest as reduced macular volume or inner retinal thinning, potentially indicating early neural compromise or developmental variation. Taken together, the pronounced thinning observed in individuals with Alzheimer’s disease and the directionally consistent, though attenuated, changes in individuals with mild cognitive impairment suggest a severity-dependent gradient, supporting the concept of a continuum of disease-related RNFL thinning.

Our result is supported by a recent study that showed an association between outer retinal changes rather than RNFL with cognitive function in middle-aged individuals with healthy retinas [13].

In the absence of macular pathology, variations in macular thickness are common across gender, age, race, and refractive error and are not typically associated with clinically significant visual functional changes. Thus, the average macular retinal thickness is not related to the degree of myopia specifically but is related to anatomical changes caused by globe elongation or high axial length [14]. The preserved visual acuity in all subjects in our study suggests that structural retinal changes do not reflect functional visual impairment and are present in individuals with intact subjective vision. This is also consistent with the finding that the average thickness was different between females and males in our study, which was not reflected in any difference in visual acuity or cognitive performance. It is also consistent with the previously reported normal thinner retinal thickness in females compared to males [15].

Oculomics describes the systematic analysis of retinal imaging features to investigate systemic disease. Given the shared embryological origin and neurovascular characteristics of the retina and central nervous system, oculomic approaches have been increasingly applied to neurological and neurodegenerative conditions. They may serve as a convenient window into brain health, especially for screening, monitoring, and predicting the risk of central nervous system pathology [16].

Changes in retinal thickness related to cognitive function were significantly associated with changes in brain structure and cerebral blood supply, indicating an association between the retina as a biomarker of brain function, including cognition [5]. Since the fovea, represented by the central circle in the Early Treatment Diabetic Retinopathy Study (ETDRS) map, has a disproportionately large representation in the primary visual cortex (V1), subtle changes in this area may reflect alterations in neural integrity [17]. The inner parafoveal layers (e.g., GCL and IPL) showed stronger associations with cognitive performance, aligning with previous literature suggesting that these layers are more susceptible to neurodegenerative processes. Interestingly, outer retinal layers, such as the ONL, which are generally thought to reflect photoreceptor integrity, have been linked to cognitive differences in aging and autism spectrum disorder (ASD), with ONL thinning observed in the temporal parafoveal area [18].

Macular thickness in our study was positively correlated with cognitive function, which is an essential morphological finding with great clinical significance. This suggests that the retina could serve as a biomarker and be a sensitive indicator of cognitive health, making it a fast, cheap, and readily available screening tool for patients and the general population. Assessing the outer retinal layers, but not the retinal nerve fiber layer, can serve as a biomarker of cognitive impairment. Although as a standalone predicting tool, OCT has not reached high sensitivity or specificity, the use of deep learning algorithms has helped increase the accuracy of this diagnostic tool to an area under the curve [AUC] = 0.91 in detecting Alzheimer’s disease in Asian populations [19].

Our results also demonstrate OCT’s ability to differentiate between mild cognitive impairment and normal cognitive function, consistent with previous reports [20]. The relationship between the retinal and cognitive physiology has been tested at the molecular level [2,3]. A Disintegrin and Metalloproteases 10 (ADAM10) is crucial for normal retinal development and is decreased in retinal degenerative disorders. A healthy retina is essential for retinoic acid production, which is crucial for ADAM10 synthesis [21]. Thus, ADAM10 is considered a biomarker for cognitive decline, which shows reduced levels in dementia and Alzheimer’s disease [22]. Other studies reported this relationship through specific neural pathways involving neurotransmitters or the microvasculature of the brain and retina, which affect oxygenation and nutrition to the retina [23].

Whether the relationship is traced to the molecular, chemical, or morphological level, clinically, there is strong evidence of this relationship, which warrants further research to validate macular OCT as a tool for cognitive testing, a biomarker of the level of cognitive impairment, and an early indicator of cognitive impairment, particularly in nonverbal patients in whom traditional cognitive screening is challenging.

People with mild cognitive impairment show minor cognitive symptoms that do not affect their daily life activities, which makes it difficult to diagnose until the symptoms advance and affect their quality of life [24]. In our study, we found that individuals with mild cognitive impairment exhibit significant thinning of macular thickness compared to those with normal cognitive performance, a striking finding that warrants further study, as mild cognitive impairment is a risk factor for future dementia. As OCT technology is now widely available in most ophthalmic clinics and increasingly used in routine eye screening, these findings suggest that retinal imaging may complement existing screening programs, such as those for diabetic retinopathy, by providing additional information relevant to cognitive disorders. In this context, macular OCT could contribute to risk stratification for dementia, supporting earlier identification and intervention.

## 5. Conclusions

The physiology of cognition can be measured by retinal morphology, particularly macular thickness and volume, and is significantly associated with cognitive performance in healthy young adults. This study highlights the potential of OCT as a non-invasive, cost-effective screening tool for early detection of cognitive health. Further longitudinal studies are warranted to validate these findings and to explore the predictive utility of OCT in cognitive aging and subclinical neurodegeneration.

## Figures and Tables

**Table 1:** Mean scores of MoCA domains in the studied population.

Domain	Mean ± SD
Visuospatial	3.93 ± 1.2
Naming	3
Attention	5.04 ± 1.4
Language	2.41 ± 0.7
Abstraction	1.9 ± 0.1
Delayed Recall	3.59 ± 1.5
Orientation	5.71 ± 1.1

**Table 2:** The scores in different MoCA domains in the Mild Cognitive Impairment group and the Normal cognitive function group.

Domain	Mild Cognitive Impairment Mean ± SD	Normal Cognitive Function Mean ± SD	*p* Value
Visuospatial	3.43 ± 0.9	4.41 ± 0.7	<0.01 **
Naming	3	3	>0.05
Attention	4.42 ± 1	5.65 ± 0.5	>0.05
Language	2.42 ± 0.6	2.55 ± 0.5	>0.05
Abstraction	1.9 ± 0.2	1.9 ± 0	>0.05
Delayed Recall	2.42 ± 1.3	4.35 ± 0.9	<0.001 ***
Orientation	5.9 ± 0.1	6	>0.05

** *p* < 0.01, *** *p* < 0.001.

**Table 3:** The difference in OCT measurements between the Mild Cognitive Impairment group and the Normal Cognitive Function group.

OCT	Mild Cognitive Impairment	Normal Cognitive Function	*p* Value
Average Thickness µm	265.84 ± 16.1	279.3 ± 12.2	<0.05 *
Central Thickness µm	215.8 ± 33.5	251.9 ± 26.1	<0.05 *
Volume mm^3^	7.66 ± 0.9	9.7 ± 1	<0.001 ***
Nasal inner macula	299.27 ± 17.2	323.09 ±16.9	<0.05 *
Superior inner macula	290.73 ± 30.3	322.53 ± 15.9	<0.01 **
Temporal inner macula	280.33 ± 17.3	306.6 ± 17.2	<0.001 ***
Inferior inner macula	286.47 ± 21.7	316.16 ± 20.6	<0.01 **
Nasal outer macula	276.93 ± 19.8	295.5 ± 17.3	<0.01 **
Superior outer macula	258.80 ± 19.9	278.02 ± 22	<0.05 *
Temporal outer macula	245.8 ± 17.1	261.27 ± 17.3	>0.05
Inferior outer macula	258.20 ± 17.5	267 ± 21.5	>0.05
RNFL	96.27 ± 8.5	96.53 ± 9.7	>0.05

* *p* < 0.05, ** *p* < 0.01, *** *p* < 0.001, RNFL = retinal nerve fiber layer.

## Data Availability

The datasets used and/or analyzed during the current study are available from the corresponding author upon reasonable request.
